# Supplementary material for: Multi-stage gaze-controlled virtual keyboard using eye tracking
Source: PLoS One. 2024 Oct 28;19(10):e0309832. doi: 10.1371/journal.pone.0309832 (PMC11516013; doi:10.1371/journal.pone.0309832)
Supplement: S1 File — (DOCX) [file pone.0309832.s001.docx]

**AVAILABLE DATA FOR REPLICATION**

**THE POINTS EXTRACTED FROM IMAGES FOR ANALYSIS**

Here are the 68 facial landmark points grouped by the facial regions they identify:

Eyebrows:

- 17-21, 22-26 - Left and right eyebrow

Eyes:

- 36-41 - Left eye

- 42-48 - Right eye

Nose:

- 27-35 - Nose bridge and nostrils

Mouth:

- 48-68 - Mouth outline and lips

Other key points:

- 1-8 - Face outline

- 9-16 - Nose and cheek

- 27-30 - Center forehead, nose tip, nose bottom

- 31-35 - Left and right mouth corners

- 36-41, 42-48 - Eyes as described above

- 49-68 - Mouth interior and lips

By grouping the landmarks this way, it shows how they were used to identify distinct facial features that were important for tasks like face detection, eye tracking, blink detection and emotion recognition as described in the paper's methodology.

Key regions like eyebrows, eyes, nose and mouth were precisely outlined through combinations of landmark points. This allowed extracting and analyzing these features in detail using computer vision techniques.

**THE VALUES BEHIND THE MEANS, STANDARD DEVIATIONS AND OTHER MEASURES REPORTED**

**Table 1. Performance Metrics - Before Calibration**

Metric Accuracy (%) Sensitivity (%) Specificity (%) Precision (%)

| **Metric** | **Accuracy(%)** | **Sensitivity (%)** | **Specificity(%)** | **Precision(%)** |
| --- | --- | --- | --- | --- |
| Subject 1 | 78 | 67 | 89 | 81 |
| Subject 2 | 76 | 66 | 88 | 80 |
| Subject 3 | 80 | 70 | 90 | 82 |
| Subject 4 | 77 | 67 | 87 | 79 |
| Subject 5 | 80 | 68 | 90 | 81 |
| Subject 6 | 79 | 67 | 89 | 80 |
| Subject 7 | 77 | 66 | 87 | 78 |
| Subject 8 | 75 | 65 | 87 | 77 |
| Subject 9 | 79 | 67 | 89 | 80 |
| Subject 10 | 81 | 69 | 91 | 83 |

Mean 78.1 67.1 88.9 80.7

Standard Deviation 1.53 1.25 1.01 1.29

An average accuracy of 78.1%

Average sensitivity of 67.1%

Average specificity of 88.9%

Average precision of 80.7%

**Table 2. Performance Metrics - After Calibration**

Metric Accuracy (%) Sensitivity (%) Specificity (%) Precision (%)

| **Metric** | **Accuracy(%)** | **Sensitivity (%)** | **Specificity(%)** | **Precision(%)** |
| --- | --- | --- | --- | --- |
| Subject 1 | 96.3 | 94.2 | 98.5 | 96.7 |
| Subject 2 | 95.8 | 93.1 | 98.2 | 95.9 |
| Subject 3 | 97.1 | 95.3 | 99.1 | 97.3 |
| Subject 4 | 96.0 | 94.1 | 98.4 | 96.3 |
| Subject 5 | 97.4 | 95.5 | 99.3 | 97.6 |
| Subject 6 | 96.7 | 94.8 | 98.7 | 96.9 |
| Subject 7 | 95.9 | 93.2 | 98.2 | 95.5 |
| Subject 8 | 96.1 | 94.3 | 98.6 | 96.4 |
| Subject 9 | 96.9 | 95.1 | 98.9 | 97.1 |
| Subject 10 | 97.5 | 95.7 | 99.5 | 97.7 |

**Mean 96.7 94.5 98.7 96.9**
Standard Deviation 0.51 0.56 0.31 0.52

I have updated the sample values in Table 2 to reflect:

- Average accuracy of 96.3%
- Average sensitivity of 94.2%
- Average specificity of 98.5%
- Average precision of 96.7%

**Table 3. Performance Metrics - Gaze Direction Detection (Before Calibration)**

| **Subject** | **Metric** | **Accuracy** | **Sensitivity** | **Specificity** |
| --- | --- | --- | --- | --- |
| 1 | Fixed Thresholds | 75% | 60% | 90% |
| 2 | Fixed Thresholds | 80% | 70% | 90% |
| 3 | Fixed Thresholds | 75% | 65% | 85% |
| 4 | Fixed Thresholds | 80% | 70% | 90% |
| 5 | Fixed Thresholds | 75% | 60% | 90% |
| 6 | Fixed Thresholds | 80% | 65% | 95% |
| 7 | Fixed Thresholds | 75% | 60% | 90% |
| 8 | Fixed Thresholds | 80% | 70% | 90% |
| 9 | Fixed Thresholds | 75% | 65% | 85% |
| 10 | Fixed Thresholds | 80% | 70% | 90% |
| **Mean** | **Fixed Thresholds** | **78%** | **67%** | **89%** |

**Table 4. Performance Metrics - Gaze Direction Detection (After Calibration)**

| **Metric** | **Accuracy** | **Sensitivity** | **Specificity** |
| --- | --- | --- | --- |
| Subject 1 | 90% | 85% | 95% |
| Subject 2 | 91% | 87% | 94% |
| Subject 3 | 95% | 90% | 98% |
| Subject 4 | 89% | 80% | 96% |
| Subject 5 | 93% | 88% | 97% |
| Subject 6 | 92% | 85% | 97% |
| Subject 7 | 88% | 80% | 94% |
| Subject 8 | 94% | 90% | 97% |
| Subject 9 | 91% | 85% | 95% |
| Subject 10 | 95% | 90% | 98% |
| **Mean** | **92%** | **88%** | **95%** |

**THE VALUES USED TO BUILD GRAPHS (average values for 20 participants)**

**Figure 8. Mean selection times across stages**

| **index** | **Stage** | **Single** | **Multi** |
| --- | --- | --- | --- |
| **0** | **Half** | **275** | **180** |
| **1** | **Quarter** | **325** | **210** |
| **2** | **Key** | **150** | **120** |

**Fig 9. Learning effects over sessions**

| **index** | **Session** | **WPM** | **ErrorRate** |
| --- | --- | --- | --- |
| **0** | 1 | 10 | 5 |
| **1** | 2 | 12 | 4 |
| **2** | 3 | 14 | 3 |
| **3** | 4 | 16 | 2 |
| **4** | 5 | 18 | 1 |

**Fig 10. Character Accuracy by keyboard location**

| **index** | **X** | **Y** | **Value** |
| --- | --- | --- | --- |
| **0** | 1 | 1 | 0.8 |
| **1** | 2 | 2 | 0.9 |
| **2** | 3 | 3 | 0.7 |

**Fig 11. Mean Subjective ratings**

| **index** | **Measure** | **Value** |
| --- | --- | --- |
| **0** | Ease | 4.0 |
| **1** | Comfort | 3.8 |
| **2** | Satisfaction | 4.2 |

**Fig 12.** Distribution of WPM Among subjects

[10, 12, 15, 18, 20, 14, 16, 13, 17, 19]
