# Supplementary material for: Multi-stage gaze-controlled virtual keyboard using eye tracking
Source: PLoS One. 2024 Oct 28;19(10):e0309832. doi: 10.1371/journal.pone.0309832 (PMC11516013; doi:10.1371/journal.pone.0309832)
Supplement: Attachment — Submitted filename: Response to Reviewers.docx [file pone.0309832.s002.docx]

Verdzekov Emile Tatinyuy

Student POSPAS

[verdzekov.emile@uniba.cm](mailto:verdzekov.emile@uniba.cm)

+237652476160

22/06/2024

Professor Laura Morett
Academic Editor
PLOS ONE

Dear Professor Laura,

Thank you for the opportunity to address the reviewers' comments and concerns regarding our manuscript "*Multi-Stage Gaze-Controlled Virtual Keyboard Using Eye Tracking*." We appreciate the thoughtful feedback and have carefully considered each point raised.

**Responses to Reviewer #1:**

1. Novelty of the Research: The reviewer notes that the referenced research cited in our article encompasses a broader scope than the research presented. We acknowledge that the use of eye-gaze input for human-computer interaction (HCI) is not a novel concept. However, our study aims to provide a more comprehensive comparison of eye typing via eye gaze and blink, particularly in terms of speeds, error patterns and error correction properties, which have not been extensively explored in prior work. We believe this comparative analysis offers valuable insights that can inform the design and improvement of vision-based text entry interfaces.

2. HCI Models: The reviewer suggests the inclusion of HCI models such as Fitts' Law and throughput to measure typing efficiency. We agree that these models could provide additional insights, and we will consider incorporating them in future research. For this study, however, our focus was on the comparative analysis of error patterns and correction strategies between the two interfaces, single stage and hierarchical approach which we implemented.

3. Ethical Statement: The reviewer notes the importance of including an ethical statement before proceeding with the research. We can confirm that our study received the necessary ethical approvals and that we have included the appropriate ethical considerations in the manuscript.

**Responses to Reviewer #2:**

1. Experimental Details: The reviewer requests more detailed information about the experiment, including the number of participants their characteristics (e.g., age, previous experience with eye trackers), their background, and the experimental setup. We have provided a more comprehensive description of the methodology in the revised manuscript, including the number of participants, their characteristics (e.g., age, previous experience with eye trackers), and the details of the experimental procedure.

2. Novelty and Literature Review: The reviewer notes the need for a more up-to-date literature review and a clearer articulation of the novelty of our work. We have expanded the literature review to include more recent publications in the field of gaze-based and vision-based text entry interfaces. Additionally, we have highlighted the specific contributions of our comparative analysis of error patterns and correction strategies, which we believe offer new insights to the research community.

3. Implementation Details: The reviewer expresses a need for more information about the implementation of our eye detection and tracking algorithms. We have provided additional details on the technical aspects of our approach, including the use of OpenCV and a discussion of the limitations and potential performance issues of our implementation. We have also considered the use of commercial eye trackers in future studies, as suggested by the reviewer.

4. Interface Design and Visualization: The reviewer requests more information about the interface design, including the dynamic splitting of the keyboard and the reasoning behind it.

As described in our paper, the full keyboard is initially presented as a single interface spanning 1500x1000 pixels. However, to optimize target selection across this wide area using only eye movements, we implemented a hierarchical interface that dynamically partitions the keyboard into progressively smaller regions guided by the user's point of gaze.

Specifically, when the user fixates near the center of the keyboard, it is first split into left and right halves of equal size (750x1000 pixels each). Eye tracking detects if their gaze moves left or right to determine the active half-region.

Each half is then further subdivided vertically into top and bottom quarters (375x500 pixels). By monitoring fixation location within the selected half, our algorithm determines whether to highlight the top or bottom quarter keys for input.

This recursive splitting allows the user to efficiently "zoom in" on their intended typing area in two stages - first selecting the broad side of the keyboard, then finer-grained quarter section. It optimizes interaction by reducing visual scanning distances at each step.

The keyboard partitioning is dynamically adjusted in real-time based on eye movements, avoiding static divisions that may not match the user's intended flow. Regions are highlighted to provide clear feedback on the current input scope.

Our evaluations found this hierarchical approach significantly improved selection speeds for distant targets versus a flat single-interface design. It leverages natural gaze behaviors to iteratively refine the input space.

Please let us know if any part of the interface logic or motivation requires further explanation. We are happy to expand on our reasoning and design process. Our goal was to optimize eyes-free interaction within the constraints of gaze input.

We have included additional screenshots, diagrams and a demo to better illustrate the interface and the user's view during the experiment.

5. Keyboard Layout and Functionality: The reviewer questions the choice of a 3x10 keyboard layout and suggests considering additional keyboard functions, such as capitalization, deletion, and special characters.

We chose a 3x10 layout to balance efficiency of target acquisition with familiarity for users accustomed to physical keyboards. While smaller than standard, concentrating keys in a narrower area aids selection accuracy given eye tracking limitations compared to manual input.

Early prototype evaluations supported the 3x10 layout as optimizing target density within tracking capabilities. Additional key layers accessed by dwell or blink modifiers avoided overcrowding while retaining familiar QWERTY positioning. Further refinements continue incorporating user feedback to refine functions based on tasks.

We believe this approach strikes a reasonable balance between constraints of gaze input and expectations of experienced typists. By concentrating frequently used keys and leveraging natural eye behaviors for modifiers, it facilitates efficient eyes-free text entry. Please let me know if any part of the implementation requires further clarification or justification. I'm happy to discuss our design choices in more detail.

We will address this in the revised manuscript, explaining our rationale for the selected layout and discussing how additional keyboard functionality could be incorporated in future iterations of the interface.

We appreciate the valuable feedback provided by the reviewers, and we are committed to addressing their concerns in a revised version of the manuscript. The revisions will strengthen the clarity, methodological details, and novelty of our work, ensuring that it meets the high standards of your journal.

Thank you for your consideration.

Sincerely,

Verdzekov Emile Tatinyuy

On behalf of the research team
