# Supplementary material for: Multi-stage gaze-controlled virtual keyboard using eye tracking
Source: PLoS One. 2024 Oct 28;19(10):e0309832. doi: 10.1371/journal.pone.0309832 (PMC11516013; doi:10.1371/journal.pone.0309832)
Supplement: Attachment — Submitted filename: Response to Reviewers.docx [file pone.0309832.s003.docx]

Verdzekov Emile Tatinyuy

Student POSPAS

[verdzekov.emile@uniba.cm](mailto:verdzekov.emile@uniba.cm)

+237652476160

22/06/2024

Professor Laura Morett
Academic Editor
PLOS ONE

Dear Professor Laura,

Thank you for your feedback on our manuscript. We have carefully considered the points raised by Reviewer #1 and are pleased to report the actions we have taken to address them in the revised version of our work.

**Reviewe #1 concern #1 “The Haar Cascade eye detection algorithm was developed around 2012, and currently, new techniques have been developed and tested to demonstrate greater efficiency.”**

As per your suggestion, we have conducted a comprehensive review of the latest advancements in eye detection algorithms, focusing on deep learning-based techniques.

We have implemented and evaluated the performance of state-of-the-art CNN-based eye detection models, such as YOLO (You Only Look Once), ResNet (Residual Network), SSD (Single Shot MultiBox Detector) and VGG (Visual Geometry Group), and compared their accuracy, robustness, and flexibility to the Haar Cascade approach used in the original manuscript.

The results of this evaluation have been incorporated into the revised manuscript, demonstrating the enhanced reliability and adaptability of our multi-stage gaze interaction technique through the use of these more advanced eye detection algorithms.

**Reviewe #1 concern #2 “The gaze point calculation algorithm employed by this research may lack flexibility across different levels of gaze planes.”**

Our work investigates several gaze point calculation algorithms and assesses the multi-stage hierarchical strategy to tackle this problem. We have evaluated our approach's adaptability and flexibility in various gaze planes and situations. The outcomes show that even in a variety of situations, the multi-stage approach greatly increases interaction efficiency when paired with cutting-edge gaze tracking algorithms. These algorithms will be further improved in the future to increase their resilience and versatility.

By applying a threshold segmentation, we separated sclera pixels from others. By counting the white pixels in the left and right halves, we were able to calculate a” gaze ratio” metric. The accuracy of gaze was determined from deep learning-based methods of face detection which we adopted. We are working on a similar paper for an eventual publication; to solve the problem of lack of flexibility in gaze point calculation algorithm employed in this research across different levels of gaze planes, our research is considering the following approaches:

·         **Adaptive Gaze Mapping**: Implementing an adaptive gaze mapping algorithm that can dynamically adjust the mapping between the user's gaze input and the on-screen keyboard regions. As the user navigates through the different levels of the hierarchical interface, the gaze mapping could be refined to provide better accuracy and responsiveness.

·         **Multi-Modal Fusion**: Combine the eye gaze input with other modalities, such as head pose or hand gestures, to improve the overall robustness and flexibility of the gaze point calculation. This could help compensate for the potential inaccuracies or inconsistencies in the gaze input across different levels of the interface.

·         **Calibration Refinement**: Implement a more advanced calibration procedure that can be performed not only at the start of the interaction but also periodically throughout the multi-stage selection process. This could help maintain accurate gaze point calculation as the user's eye movements and interaction patterns evolve.

·         **Predictive Modeling**: Develop predictive models that can anticipate the user's intended target based on their gaze patterns and the current context within the multi-stage interface. This could help refine the gaze point calculation and reduce the impact of potential errors or inconsistencies.

·         **Feedback and Correction Mechanisms**: Provide visual, auditory, or haptic feedback to the user to indicate the accuracy of the gaze point calculation and allow for real-time corrections or adjustments. This could help the user adapt their interaction patterns to improve the overall performance of the gaze point calculation.

By incorporating these strategies, our research can enhance the flexibility and robustness of the gaze point calculation algorithm, ensuring consistent and accurate performance across the different levels of the multi-stage hierarchical interface.

**Reviewe #1 concern #3 “The design of the virtual keyboard layout, including its size and position, must be developed in accordance with the pointing capabilities of the device. The process of determining the appropriate size and interface format for eye-tracking has not yet been tested with diverse samples prior to its development and the subsequent measurement of the eye-tracking tool's effectiveness in this research.”**

We are grateful for this insight and have moved to resolve the issues raised. As part of our investigation, we assess the virtual keyboard arrangement in terms of its alignment with the device's pointing capabilities by varying its sizes and placements. To evaluate the multi-stage gaze-controlled keyboard's efficacy, we ran tests on a wide range of users. The outcomes show that the suggested design is efficient and easy to use, with notable gains in accuracy and speed of selection over single-step methods.

By addressing these points in the revised manuscript, we have demonstrated our commitment to strengthening the technological foundations, design choices, and target user considerations of our multi-stage gaze-controlled virtual keyboard approach. We believe that incorporating your valuable suggestions has enhanced the overall quality and impact of our research.

Thank you again for your thorough review and constructive feedback. We are confident that the revised manuscript will present a more comprehensive and robust evaluation of our work.

Thank you for your consideration.

Sincerely,

Verdzekov Emile Tatinyuy On behalf of the research team

[Verdzekov.emile@uniba.cm](mailto:Verdzekov.emile@uniba.cm)

+237652476160
